# A Review of Recent Patents on the Protozoan Parasite HSP90 as a Drug Target

**DOI:** 10.2174/1872208311307010002

**Published:** 2013-04

**Authors:** Sergio O Angel, Mariana Matrajt, Pablo C Echeverria

**Affiliations:** 1 Laboratorio de Parasitología Molecular, IIB-INTECH, CONICET-UNSAM, Av. Intendente Marino Km. 8.2, (B7130IIWA), Chascomús, Prov. Buenos Aires, Argentina; 2 Department of Microbiology and Molecular Genetics, University of Vermont, Burlington, VT 05405, USA; 3 Département de Biologie Cellulaire Université de Genève Sciences III, Geneva, Switzerland

**Keywords:** Plasmodium, Toxoplasma, HSP90, geldanamycin, 17-AAG, patent, therapy

## Abstract

Diseases caused by protozoan parasites are still an important health problem. These parasites can cause a wide spectrum of diseases, some of which are severe and have high morbidity or mortality if untreated. Since they are still uncontrolled, it is important to find novel drug targets and develop new therapies to decrease their remarkable social and economic impact on human societies. In the past years, human HSP90 has become an interesting drug target that has led to a large number of investigations both at state organizations and pharmaceutical companies, followed by clinical trials. The finding that HSP90 has important biological roles in some protozoan parasites like *Plasmodium* spp, *Toxoplasma gondii* and trypanosomatids has allowed the expansion of the results obtained in human cancer to these infections. This review summarizes the latest important findings showing protozoan HSP90 as a drug target and presents three patents targeting *T. gondii*, *P. falciparum* and trypanosomatids HSP90.

## 1. INTRODUCTION

Protozoan parasites include a very diverse group of unicellular eukaryotic organisms from the kingdom Protista. The most common infections in humans from this group are caused by *Plasmodium spp*. and *Toxoplasma gondii* (both from the phylum Apicomplexa), as well as by the trypanosomatids *Trypanosoma* and *Leishmania spp. *Protozoan parasites are responsible for considerable mortality and morbidity, affecting more than 500 million people worldwide [1]. The epidemiological control of these parasites is still unsatisfactory due to the difficulties to control both the insect vector and/or the human/animal reservoirs for the different parasitoses. Although synthetic vaccines against different protozoan parasites have been developed, the results obtained so far have demonstrated that such strategy is a difficult task. Thus, chemotherapy remains an essential component of both clinical management and disease control programs. Although there are efficient therapies against almost all of the protozoan pathogens, a large number of factors, such as their high cost, poor patient compliance, drug resistance, low efficacy and poor safety, limit their utility. As a consequence, the search for new drugs against protozoan parasites is constantly needed [1]. 

Malaria is a mosquito-borne infectious disease of humans and other animals caused by unicellular obligate intracellular protozoan parasites of the genus *Plasmodium*. Malaria is currently endemic in the tropical and sub-tropical zones of Asia, Africa, South and Central America, but also constitutes a serious problem for travelers as well as for people working in endemic regions. Nearly half of the world’s population (3.3 billion people) is at risk in more than 100 countries [2]. A recent study has estimated that 1,238,000 people died from malaria in 2010 [3]. Five primary species of malaria parasites infect humans: *P. falciparum*, *P. ovale*, *P. malariae*, *P. vivax *and *P. knowlesii*. The severe disease is mainly caused by *P. falciparum *whereas *P. vivax *causes the majority of malaria morbidity outside Africa. The clinical manifestations of malaria include fever, shivering, arthralgia (joint pain), vomiting, jaundice, hemoglobinuria, convulsions, metabolic alterations, renal failure, liver and lung dysfunctions, anemia and cerebral malaria (coma) [4].

*Toxoplasma gondii *is an obligate intracellular parasite and the causative agent of toxoplasmosis, a worldwide infection affecting 500 million to 1 billion people in a chronic asymptomatic form [5]. In humans, this parasite replicates asexually in the form of rapidly growing ‘tachyzoites’ and latent ‘bradyzoite’ tissue cysts. Tachyzoites are responsible for acute illness and congenital neurological birth defects, while the more slowly dividing bradyzoite form can remain latent within the tissues for many years, and remains capable of switching to damaging tachyzoites if host immunity fades. During the first few weeks of infection, toxoplasmosis is either asymptomatic or causes a mild flu-like illness. However, those with a weakened immune system, such as AIDS patients, pregnant women or newborns with a congenital infection, may become seriously ill, and occasionally die. The parasite can cause encephalitis (inflammation of the brain) and neurologic diseases, and can affect the heart, liver, inner ears, and eyes (chorioretinitis). Recent research has also linked toxoplasmosis with brain cancer, attention deficit hyperactivity disorder, obsessive compulsive disorder, and schizophrenia [6-10]. The present chemotherapy for toxoplasmosis is still deficient. The anti-*T. gondii* therapy is not well tolerated by individuals with AIDS, and is efficient only against the tachyzoite stage, but not against bradyzoites. It is thus important to develop new and safer drugs [11].

*Leishmania spp*, *Trypanosoma cruzi* and *Trypanosoma brucei*, all of which are transmitted by an insect vector (the sand and tsetse flies are the vectors of *Leishmania* spp and *T. brucei* respectively, whereas blood-sucking insects of the subfamily *Triatominae* are the vectors for *T. cruzi*), are the most important trypanosomatids associated with human health. Human leishmaniosis is caused by about 21 of 30 species, among which are the *L. donovani* complex with three species (*L. donovani*, *L. infantum*, and *L. chagasi*) and the *L. mexicana* complex with three main species (*L. mexicana, L. amazonensis, *and* L. venezuelensis*). Cutaneous leishmaniasis is the most common form of leishmaniasis, whereas visceral leishmaniasis (VL) is a severe form in which the parasites have migrated to vital organs. Each year 500,000 new human cases of VL are reported [12]. The trypanosomatid biological cycles alternate between the amastigote form in the vertebrate host and the promastigote form in the gut of the sandfly vector. Chagas disease represents a serious health problem in Latin America, with an overall prevalence of about 12-16 million cases [13]. In humans, its acute phase usually causes no more than local swelling at the site of infection. In the chronic phase, almost all of the infected individuals remain asymptomatic, but near 30-40% of them may develop clinical symptoms characteristic of this phase, such as cardiac and or digestive alterations. The existing chemotherapy, based on benznidazole and nifurtimox, shows substantial toxicity, variable effect on different parasite stocks and poor activity on the chronic phase [14]. *T. brucei* is responsible for the vector-borne disease named Human African Trypanosomiasis (HAT) or sleeping sickness. The WHO estimates that as many as 60 million people are at risk to contract HAT [15]. This disease is 100% fatal if it is not treated, and the current drug therapies have significant limitations due to toxicity and difficult treatment regimes. Therefore, it is necessary to continue developing new drugs [16]. 

In this review, we have summarized the findings on the biological role of the protozoan Heat Shock Protein 90 (HSP90), with emphasis on *Plasmodium* and *Toxoplasma* pathogens, and its value as a novel target for developing new therapies against these pathogens. In this context, we present the features of three patents that involve *Plasmodium* and *T. gondii* HSP90 as a drug target.

## 2. THE HSP90 HETEROCOMPLEX

The Heat Shock Protein (HSP) families include a large number of proteins constitutively expressed in high quantities, and whose expression increases when the cell is subjected to stress conditions [17]. Interestingly, it has been shown that HSPs have important roles in the organism, responding to environmental stress factors, and characterized by the turn on and the turn off of some genes [18]. HSPs are highly conserved within the three main phylogenetic domains (Bacteria, Archea and Eukarya) thus suggesting an important role both for them and for other molecular chaperones.

Among HSPs, eukaryotic HSP90 has a highly selective activity in stressed and unstressed cells, where it is responsible for the recovery of misfolded proteins, protein maturation, intracellular transport of proteins, and regulated activities of nuclear hormone receptors as well as other transcription factors, and protein kinases involved in signal transduction and translation control [18-20]. The function of HSP90 is highly dependent on ATP and on its ATPase activity. The benzoquinone ansamycin antibiotic geldanamycin (GA), or its derivates, binds to HSP90 by interaction with its ATP binding pocket with much higher affinity than ATP itself [21], altering the function or folding of proteins that bind to HSP90 (named client proteins), a process that leads unbound proteins to the degradation pathway [22]. In humans, many HSP90 client proteins include oncoproteins with important functions in the development and promotion of cancer, making HSP90 as an important target in cancer therapy [23]. 

The HSP90 chaperone, which is present in all protozoan parasites studied, has a high amino acid identity to its human ortholog. In some cases, it has been shown to have an expression pattern linked to parasite development [24-27]. Recent efforts to decipher the interactome networks of *T. gondii* and *P. falciparum* HSP90 have shown the presence of several HSP90-interacting proteins in common with higher eukaryotes (mainly those related to ATP generation, protein and nucleic acid metabolism) [28, 29]. Moreover, the broad range of functions regulated by chaperones seems to involve other chaperones, chromatin-associated proteins or protein trafficking. Interestingly, proteins related to cytoadherence or *T. gondii*-specific kinases involved in the host-parasite interaction, as well as hypothetical proteins, have also been identified as specific interactors of *P. falciparum* and/or *T. gondii* HSP90. Furthermore, “conserved” client proteins might not fulfill the same cellular roles between protozoan and other eukaryote cells. Thus, based on the importance and conserved mechanism with the human counterpart, it is reasonable that drugs against protozoan parasite HSP90 will benefit from the development of anti-HSP90 therapy against cancer. 

## 3. GELDANAMYCIN AND ITS DERIVATIVES BLOCK PARASITE DIFFERENTIATION AND GROWTH: HSP90 AS A NOVEL DRUG TARGET 

The high conservation among HSP90 and co-chaperones of different organisms is evidenced not only in its amino acid sequence, but also in its susceptibility to GA. GA is a benzoquinone ansamycin Fig. (**1**), that has been shown to bind directly to HSP90 and interfere with the HSP90-client protein heterocomplex formation [30]. This compound was first isolated from *Streptomyces hygroscopicus* in 1970, as a new antibiotic with moderate *in vitro* activity against protozoa, bacteria and, fungi as well against L-1210 (mouse lymphocytic leukemia cells) and KB (cell line derived from a human carcinoma of the nasopharynx) cells growing in culture [31]. In 1994, Whitesell *et al.* [32], patented its use as a tumoricidal drug (Table **1**). The mechanism of action of GA on HSP90 is to inhibit the ATPase activity of the chaperone by competing with ATP for binding to the N-terminal domain nucleotide binding pocket [33]. The inhibition interferes in the maturation process of client proteins, facilitating their ubiquitin-mediated proteasomal degradation [34]. However, GA has shown to be of poor value as a drug because of its high toxicity (poor solubility and significant hepatotoxicity in animals) and *in vivo* instability [23]. For this reason, numerous efforts have been made to develop GA semisynthetic analogs, such as 17-allylamino-17-demethoxygeldanamycin (17-AAG) and retaspimycin hydrochloride (IPI-504), a water-soluble hydroquinone hydrochloride salt derivative of 17-AAG Fig. (**1**), which have been subjected to phase I, II and III clinical trials with varied results [35]. Although 17-AAG is less toxic than GA, it still has some limitations such as poor solubility and liver toxicity, the latter attributable to the benzoquinone [36]. In this regard, in the retaspimycin hydrochloride compound, the quinone was reduced to hydroquinone, which arose as a more potent inhibitor of HSP90 [37]. In addition to ansamycine derivatives, there are several drugs targeting HSP90 that are in study for cancer therapy, such as purine and purine-like analogs, coumarin-based inhibitors (novobiocin), dihydroindazolone derivatives, heterocyclic Amines, radicicol (resorcinol-bearing compound) and analogs [23, 35]. 

In fungi, HSP90 has been shown to be crucial for resistance to azoles and echinocandins, via the client protein calcineurin [38-40]. Pharmacological inhibition of HSP90 or calcineurin reduces echinocandin tolerance *in vitro* [40]. Moreover, infection of mice with *Candida albicans* under genetic impairment of *hsp90* gene expression significantly reduces kidney fungal burden, and enhances the efficacy of echinocandin [41]. Human recombinant monoclonal antibody against HSP90 plus lipid-associated amphotericin B caused significant clinical and culture-confirmed improvement in the outcome of patients with invasive candidiasis [41]. These studies support HSP90 as an interesting drug target for cancer and pathogens. In this sense, there are several *in vitro* and *in vivo* studies analyzing the effect of GA and its derivatives on the life cycle of protozoan parasites. 

### 3.1. *In Vitro* Studies

The most direct evidence of the interaction between GA and protozoan parasite HSP90 has been recently shown by Pallavi *et al.* [42]. These authors demonstrated that GA binds to *P. falciparum* HSP90 with high affinity, presenting a dissociation constant of GA for PfHsp90 of 1.05 µM, whereas its value with human HSP90 was of 4.4 µM. Moreover, the inhibition constant IC50 of HSP90 ATPase activity due to GA for *P. falciparum* HSP90 was three times lower than that for human HSP90 indicating that parasite HSP90 is more sensitive to GA-mediated inhibition. Similar results were obtained in the same work with *Trypanosoma evansi* HSP90 protein [42]. 

Before these studies, numerous studies had demonstrated the ability of GA to block protozoan parasite development. The life cycle of *Leishmania* *spp* involves sandflies as a vector and humans and other animals as definitive hosts. When the parasites are injected from the sandflies to mammals, they undergo a significant rise in the environmental temperature. Noteworthy, the *in vitro* mimicking of the temperature shift suffered by promastigotes when transmitted to mammal hosts has shown a transient increase of the synthesis of HSPs that are post-transcriptionally up-regulated [43-45]. In 2001, Wiesgigl and Clos [27] observed that the inactivation of *L. donovani* HSP90 by GA induces growth arrest in the G2 Phase of the cell cycle of the promastigote stage as well as the differentiation from the promastigote to the amastigote stage. It has been also demonstrated that *L. donovani* promastigotes exhibit apoptotic morphological changes after GA treatment at a high temperature [46]. The treatment of different *T. cruzi* stages with GA has demonstrated that HSP90 is essential for parasite cell cycle control, in which treated epimastigotes and blood-form trypomastigotes show G1 arrest. However, in these cases, the inhibition of HSP90 did not induce parasite differentiation [25]. 

The treatment of *P. falciparum* (synchronous ring stage) with GA inhibits its growth, measured as parasitemia, and also the progression from the ring stage to trophozoite [24]. However, in that study, the authors showed that the transition from trophozoite to schizonts and the reinvasion of new erythrocytes were less significantly affected. In order to detect small molecules that specifically target malaria HSP90, Shahinas *et al.* [47] analyzed three libraries consisting of natural compounds, FDA-approved drugs and pharmacologically active compounds consisting of approximately 4000 small molecules for competitive inhibition of the ATP-binding (GHKL) domain of *P. falciparum* HSP90. Three compounds, (2R)-2-amino-3-phosphonopropionic acid, harmine (harmaline) and acrisorcin Fig. (**1**), were detected to inhibit specifically PfHSP90 and to act synergistically with chloroquine. 

The treatment of *T. gondii* tachyzoites with GA blocks host cell invasion and parasite replication [48]. Echeverria *et al.* [26] showed that the addition of GA to parasite cultures blocks tachyzoite to bradyzoite and bradyzoite to tachzyoite transition. Once bradyzoites become tachyzoites, HSP90 localization changes and is excluded from the bradyzoite nucleus, being present only in the cytoplasm of the tachyzoite stage [26]. All these findings support the protozoan HSP90 as a novel antiparasitic target.

### 3.2. *In Vivo* Studies

The malaria field is where the principal advances have been made in using *in vivo* experiments to demonstrate the efficacy of GA derivatives (see [42-43] for reviews). Two *in vivo* experiments have demonstrated the efficacy of GA derivatives. Besides demonstrating the binding of GA to *P. berghei* HSP90, Pallavi *et al.* [42] used the GA analog 17-AAG to treat infected mice. Their results are highly promising: while the parasitemia of untreated infected mice rose steadily, peaking at 80-90% until the death of the animal, the parasitemia of drug treated-mice was significantly attenuated. In agreement with the parasitemia levels, on day seven post-infection, all the untreated infected animals died, whereas on day 21 post-infection near 50% of 17-AAG-treated animals survived. The authors extended the pre-clinical study of 17-AAG to *T. evansi*, another protozoan parasite, which causes surra in animals. Once again, the untreated animals died after infection whereas those that were treated with 17-AAG showed no parasitemia, resulting in 60% survival [42]. More recently, 17-AAG and 17-N-(3-(2-(-2(3-aminopropoxy) ethoxy)propyl)pent-4-ynamide-17-demethoxygeldanamycin (17-PEG-Alkyn-GA) Fig. (**1**), a highly water soluble pegylated derivative of GA, were tested against a murine model infection with* P. yoelii* [49]. The drug was inoculated on day 6 after infection, when malaria symptoms were evident. In control groups, the parasitemia reached almost ~60% and all the animals died by day 14 postinfection. The administration of 17-AAG or 17-PEG-Alkyn-GA on day 6 post-infection resulted in control of parasitemia. However, a second dose was needed for complete clearance of the parasites and cure of mice [49]. In this case, by using either 17-AAG or 17-PEG-Alkyn-GA, three out of four mice survived the experiment. In addition, the anti-HSP90 treatment caused a shift in parasite invasion specificity, from normocytes, which are lethal for *Plasmodium*, to more benign reticulocytes, expanding the persistence of parasitemia for a longer period compared to cloroquine treatment. This long persistence before parasite elimination could result in the development of immunity to subsequent challenges of *P. yoelii*. 

While it was demonstrated that GA interferes with the development of *Leishmania*, *Toxoplasma* and *Trypanosoma in vitro*, its efficacy at pre-clinical level has not yet been addressed. 

## 4. FROM THE BIOLOGICAL ROLE TO PATENTS

Based on the highly interesting results obtained with GA and its derivatives against protozoan parasites infection, and on the increased development of anti-HSP90 drugs due to anti-cancer therapy, three inventions targeting parasite HSP90 have been patented (Table **1**). Two of them involve this chaperone for the treatment of toxoplasmosis and malaria, whereas the third invention involves applications to cancer but also to *P. falciparum*, *L. donovani* and *T. cruzi*.

### 4.1. US20117968096 [50]

Patent 20117968096 is based on our discovery that inhibitors of the protein HSP90 block *T. gondii* differentiation, in particular the conversion from bradyzoite cysts (responsible for the chronic infection) to the active replicative stage (responsible for the acute infection) [26]. Therefore, this invention can be used to treat the latent infection. Since all the drugs currently available in the market are against the active replicative stage, this patent represents the first step towards developing drugs for the chronic infection. Such drugs would prevent reactivation, which can have devastating effects on immunocompromised patients. In addition, since some HSP90 inhibitors are under clinical trials for cancer chemotherapy and these patients have a great chance to reactivate toxoplasmosis, these drugs could be used for the dual purpose of cancer treatment and prevention of toxoplasmic reactivation. 

The patent specifies a group of inhibitors of HSP90 such as benzoquinone ansamycin derivatives besides GA that can be applied for anti-HSP90 therapy during latent infection: 7-allylamino-1-deoxy-geldanamycin, 17-AAG, 17-dimethylaminoethylamino-geldanamycin (17DMAG),17-(3-(4-maleimidobutyrcarboxamido)propylamino)-17-demethoxy-geldanamycin (17-GMB-APA-GA). The invention also involves a screening method for identifying compounds for treating latent *T. gondii* infection, wherein the reference compound is an HSP90 inhibitor. Briefly, it includes the analysis of a candidate compound against HSP90 in an *in vitro* culture of cells infected with *T. gondii* under conditions which allow stage interconversion of the parasite.

### 4.2. US20097611853 [51]

This patent relates to a novel assay to screen for anti-malarial drugs using plasmodial HSP90 as a target. Based on the toxic activity of GA and 17-AAG against malaria and the evidence that GA binds *P. falciparum* HSP90 [42], the inventors suggest that every compounds that binds at a GA-site on parasite HSP90 can be a potential anti-malarial agent. The binding of GA to pathogen HSP90 has been demonstrated *in vitro*, and such binding can be quantified using suitable immunochemical, radiochemical or non-radioactive assays. Therefore, the patent relies on a method to screen anti-*P. falciparum* HSP90 compounds. Such assays can be further developed into high throughput assays using the currently known technologies. The method includes the parasite preparation, reaction of the parasite lysate with immobilized compounds, which are covalentely linked to a matrix from the group consisting of agarose and carboxymethylated dextran, and the reaction of saponin-free plasmodial trophozoite lysate. The detection is done by Western blot using polyclonal antibodies to PfHsp90. An alternative strategy is to use radiolabeled trophozoite protein extracts followed by the detection of PfHSP90 by phosphorimager analysis. In addition, a non-radioactive assay (surface plasmon resonance analysis with biosensor system) is also a proposal of the invention. 

The selected compounds can be further tested on parasite ring stage, analyzing the inhibition of stage progression as well as parasitemia which is estimated using giemsa staining. The patent also describes a novel methodology to analyze the effect of anti-HSP90 drugs on parasite growth by using flow cytometry analysis. In this assay, acridine-stained parasites stages (ring and trophozoites) can be identified by the analysis of scattergrams for parasites at various times after synchronization.

### 4.3. WO2007098229 [52]

Patent WO2007098229 is not specific to protozoan parasite, because it relates to novel geldanamycin derivatives which have antitumor and antiparasitic properties. The human parasites mentioned are *P. falciparum*, *T. cruzi*, and *L. donovani*, which could be susceptible to exposure to proposed geldanamycin derivatives. 

Based on the fact that the natural compound GA is too toxic for therapeutic use, the inventors suggest the requirement of GA derivatives which may be useful as anticancer agents, and may also have antiparasitic activity, and preferably having minimal human toxicity. The authors proposed several GA derivatives based on different substitutions. Noteworthy, the compounds of this invention were designed by structure-based molecular modeling and chemical synthesis. The resulting compounds are assessed for specific interaction by means of binding studies and the determination of the drug concentrations needed for killing the parasites. The inventors described different GA derivatives that can increase its interaction with different residues present in the *P. falciparum* HSP90.

## 5. CONCLUSIONS & FUTURE TRENDS

Despite the great advances observed in the use of HSP90 as a drug target in cancer research and clinical trials, a therapy based on the protozoan parasite HSP90 is in an early phase. The finding that HSP90 is a key molecule for the course of infection in different parasites should focus the attention of pharmaceutical companies to take the advantage of the experience in cancer and extend it to the infectious diseases described in this review. HSP90 inhibitors like GA have already been shown to affect more efficiently parasites than host cells. Recent efforts attempted to find even more specific anti-malarial hsp90 inhibitors [47].

The patents addressing parasite HSP90 as drug target involve different strategies of inventions inlcuding a method to identify compounds that bind PfHSP90, use of HSP90 inhibitors to treat *T. gondii* latent infection and different GA derivatives against *P. falciparum* and trypanosomatid HSP90. The *in vivo* analysis suggests that the anti-HSP90 treatment could have an anti-parasitic effect, at least for *Plasmodium spp* and *T. evansi* [42, 49]. Therefore, it is reasonable to expect future studies that involve animal models of malaria, Chagas, leishmaniosis and toxoplasmosis either using some of the compounds described above or new synthesized molecules. In addition, the role and importance of *T. brucei* HSP90 and the effect of HSP90 inhibitors during parasite infection are still intriguing. 

In the case of *T. gondii*, severe toxoplasmosis can occur in newborns with congenital toxoplasmosis or immunodeficient patients (HIV patients, transplanted patients, etc). The invention US20117968096 addressed the treatment of the reactivation of infection. Different scenarios are expected: cases in which the treatment is useful for a limited period (e.g. individuals under transplantation), or cases where the treatment will be required for a long period (HIV patients). Therefore, future studies should consider these different scenarios. 

One aspect that makes the anti-HSP90 therapy attractive is that this chaperone has a pleiotropic role, assisting a wide range of biological functions both in higher eukaryotes and protozoan parasites. Proteomic analysis suggests that *Toxoplasma* is not the exception [28]. The treatment of extracellular parasites with GA has shown to block tachyzoite host cell invasion [48]. Therefore, the *in vivo* analysis of HSP90 inhibitors could show surprising results for the different parasite infection models. 

Another area of analysis is the possible use of HSP90 inhibitors in combination with other therapies. The use of different small molecules specific for *Plasmodium* HSP90 in combination with chloroquine has shown to have a synergistic effect, serving as a strong basis for combination therapy in human disease [47]. In the case of toxoplasmosis, the combination of accepted therapy (effective against tachyzoites) with anti-HSP90 drugs (blocking the possibility to convert to the drug-protected cyst form) could be effective to maximize the parasite clearance of the host, and it may prevent or reduce latent infections.

It is also expected that in the upcoming years novel drugs that bind the protozoan HSP90 will be discovered and that these compounds could potentially be used in clinical or pre-clinical trials. 

## Figures and Tables

**Fig. (1):**
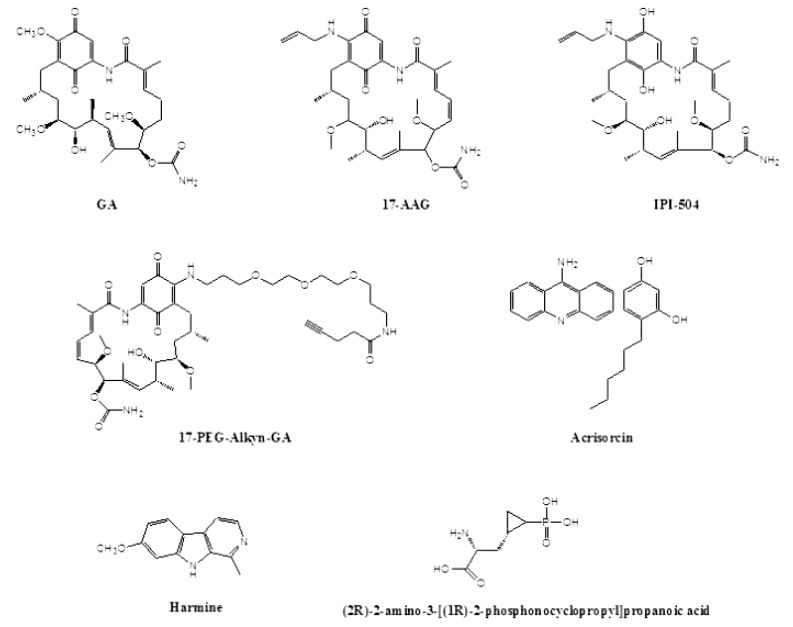
Structure of different anti-HSP90 inhibitors.

**Table 1.** Patents that Involve Protozoan HSP90 as Drug Target.

Ref. Authors	Year	Title	Patent No.	Country Area	Applicant
[32] Whitesell, L., Neckers, L., Trepel, J., Myers, C	1994	Tumoricidal activity of benzoquinonoid ansamycins against prostate cancer and primitive neural malignancies	WO9408578	World WIPO	Conforma Therapeutics Corp [US]; Zhang Lin [US]; Le Brazidec Jean-Yves [US]; Boehm Marcus F [US]; McHugh Sean Konrad [US]; Fan Junhua [US]; Fritz Lawrence C [US]; Burrows Francis J [US]
[50] Matrajt, M. L., Angel, S. O., Echeverria, P. C.	2011	Methods and compositions for treating *Toxoplasma*	US20117968096	United States	The University of Vermont and State Agricultural College (Burlington, VT, US)
[51] Tatu, U., Pavithra, R. S., Banumathy, G.	2009	Assay to screen for anti-malarials	US20097611853	United States	Indian Institute of Science (Bangalore, IN)
[52] Wenkert, D., Kuhn, L., Scheller, E., Kron, M., Shen, Y.	2007	Geldanamycin derivatives and method of use thereof	WO2007098229	World WIPO	Michigan State University (East Lansing, MI, US)
